# Impact of systemic lupus erythematosus organ damage on unemployment or disability from a population-based cohort

**DOI:** 10.1186/ar3947

**Published:** 2012-09-27

**Authors:** SS Lim, M Agan, CM Drenkard

**Affiliations:** 1 Emory University School of Medicine, Atlanta, GA, USA

## Background

Systemic lupus erythematosus (SLE) predominantly develops in younger age groups, when many are establishing themselves in the workforce. The development of a chronic, autoimmune condition during this period can have a devastating impact on employment. The objective was to determine the impact of different organ damage in patients with SLE on employment loss from a large, population-based cohort.

## Methods

The source of data was from the 2011 to 2012 annual patient-reported survey of the Georgians Organized Against Lupus (GOAL) Study, an ongoing population-based cohort of patients with validated SLE in Atlanta, GA assembled primarily from the Georgia Lupus Registry (GLR). The GLR was supported by the Centers for Disease Control and Prevention and designed to more accurately estimate the incidence and prevalence of SLE. In partnership with the state health department, the GLR was able to access protected health information without patient consent. GOAL Study participants were surveyed regarding employment status at the time of survey completion along with other demographic information. Organ damage was measured using the Brief Index of Lupus Damage. Disease activity was measured using the SLE Activity Questionnaire (SLAQ). Logistic regression analysis was used to measure the association between categories of organ damage and unemployment/disability.

## Results

A total of 459 SLE patients were surveyed with a mean age of 46.5 (SD ± 10), 13.4 (SD ± 8.6) years of disease, and 14.2 (SD ± 2.8) years of education; 93.2% were female, 79.5% were black and 14.4% white. One hundred and ninety-seven (42.9%) were working and 262 (57.1%) were unemployed/disabled. The median duration of loss of employment was 7.1 years (IQR 3.1 to 12.3). See Table 1.

**Table 1:** Factors related to unemployment/disability in SLE

Sociodemographic factor	Univariate			Multivariate model^a^		
	
	OR	95% CI	*P *value	OR	95% CI	*P *value
Gender (female vs. male)	0.61	0.28 to 1.34	0.22	0.88	0.25 to 3.03	0.83
Race (white vs. black)	0.30	0.17 to 0.52	<0.001	0.36	0.15 to 0.86	0.021
Education (per year)	0.75	0.69 to 0.81	<0.001	0.75	0.67 to 0.84	<0.001
Depression	4.09	2.68 to 6.25	<0.001	4.52	2.42 to 8.45	<0.001
Currently taking corticosteroids	2.65	1.80 to 3.91	<0.001	2.06	1.18 to 3.62	0.012
Organ damage						
Cardiovascular	7.48	3.49 to 16.03	<0.001	7.78	2.77 to 21.82	<0.001
Renal	5.34	2.04 to 13.97	<0.001	9.38	2.6 to 33.85	0.001
Neuropsychiatric	2.76	1.59 to 4.81	0.001			
Neuropsychiatric, low SLAQ				6.65	1.41 to 31.44	0.017
Neuropsychiatric, high SLAQ				0.93	0.37 to 2.34	0.87

^a^Logistic regression.

## Conclusion

In total, 57.1% of SLE patients were unemployed or disabled at the time of the survey. Organ damage from SLE has a profound association with unemployment/disability. In the multivariate model, low education level and depression were independently associated with unemployment/disability. In line with other studies, cardiovascular and renal damage were associated with unemployment/disability. Previous studies have not reported disease activity (SLAQ) as a mediator and should be considered particularly when evaluating neuropsychiatric damage and its association with employment.

